# Commentary: Epidemiology, clinical features and prognostic factors of pediatric SARS-CoV-2 infection: Results from an Italian multicenter study

**DOI:** 10.3389/fped.2022.1067453

**Published:** 2022-12-09

**Authors:** Sonia Brio, Sergio Verd, Jan Ramakers, Clara Sorribes, Xavier Rodríguez-Fanjul, Ruth Díez

**Affiliations:** ^1^ Intensive Care Unit, Department of Paediatrics, Santa Creu I Sant Pau Hospital, Barcelona, Spain; ^2^ La Vileta Surgery, Paediatric Unit, Balearic Department of Primary Care, Baleares Health Service, Palma de Mallorca, Spain; ^3^ TERCIT Cell Therapy Department, Balearic Institute of Medical Research (IdISBa), Palma de Mallorca, Spain; ^4^ Department of Pediatrics, Son Espases University Hospital, Palma de Mallorca, Spain; ^5^ Intensive Care Unit, Department of Pediatrics, Joan XXIII Hospital, Tarragona, Spain; ^6^ Intensive Care Unit, Department of Paediatrics, Germans Trias i Pujol Hospital, Barcelona, Spain

**Keywords:** neonate, breastfeeding, human milk, COVID-19, kawasaki disease

**A Commentary on:**
Epidemiology, clinical features and prognostic factors of pediatric SARS-CoV-2 infection: results from an Italian multicenter study By Brio S, Verd S, Ramakers J, Sorribes C, Rodríguez-Fanjul X, Díez R. (2022). Front. Pediatr. 10:1067453. doi: 10.3389/fped.2022.1067453

To the Editor,

This insightful paper by Grazzino et al. (1) on paediatric characteristics and risk factors for disease progression of COVID-19 investigates, among other important issues, the possibility that the wide clinical variation observed in this disease may be explained by factors involved in the early stages of life. They point out that ex-prematurity and young age were risk factors for hospital admission, and that viral co-infections predict severe disease. We appreciate the herculean effort undertaken to summarize data from one of the largest European cohorts on paediatric COVID-19. However, we are left with the question of whether a very important aspect of the natural history of SARS-CoV-2 paediatric infection is still missing.

Early after the onset of this pandemic, it was stressed that previous or current optimal nutrition might mitigate the risk and morbidity associated with COVID-19 (2). As hospital-based or primary care paediatricians, and breastfeeding champions, we would like to focus on whether consumption of mother's milk confers benefits against COVID-19 years after lactation is terminated. The influence of breast milk on the development of infectious diseases later in life has been under study for several decades. Early research demonstrated that infants who were breastfed have increased thymus size than those who were formula-fed, and there is a correlation between breastfeeding and CD8 + T cells (3, 4). In addition, breastfeeding is associated with a decreased risk for wheezing (5), otitis (6), H pylori colonization (7), Hib infection (8), appendectomy (9), and tonsillectomy (10) in the medium term. Correspondingly, the odds of young schoolchildren having more than one sick visit per year are significantly associated with the duration and exclusivity of breastfeeding (11).

Human milk feeding triggers lifetime immune-protective responses by the host through various mechanisms. Among others, mucosal maturation, immune modulation, microbiota shaping, or balance between pro-inflammatory and antioxidant status contribute to the antiviral activity of human milk (12).

A study on the UK Biobank population cohort aimed to identify associations between early life factors, including breastfeeding, and the risk of COVID-19 infection and hospitalization. The longitudinal UK Biobank cohort includes more than half a million participants aged 37–73 years old when recruited in 2006–2010. Early-life variables were self-reported, and respondents were categorized as breastfed if they confirmed that they were breastfed when they were babies. Of all eligible respondents, 43,428 had been COVID-19 tested, 7,733 had tested positive and 2,494 were hospitalized due to COVID-19. A multivariate logistic model showed that respondents who were breastfed had 12% lower odds of contracting COVID-19 in the first peak just as much as in the second peak of this pandemic. Breastfeeding was negatively associated with hospitalization only in the first peak (13).

On a much smaller scale, our practices in the community or hospital settings embraced the opportunity to perform targeted surveys to check whether the previous breastfeeding prevents SARS-CoV-2 colonization or the Multisystem Inflammatory Syndrome in Children associated with COVID (MIS-C). For this purpose, during the second peak of the COVID-19 pandemic, we analyzed the effect of any breastfeeding on the risk of testing positive for SARS-CoV-2 among 691 Majorcan children (mean age = 67.8 months) attending community or hospital emergency services. We found that approximately 1 in 60 ever-breastfed children had tested positive for SARS-CoV-2, at odds with 1 in 25 never breastfed children (OR, 2.48; *P *= 0.036) (14). Hence, it was proven that initially breastfed children in our area remained at lower risk of COVID-19 during August–December 2020 than exclusively previously formula-fed children. However, a difficulty with breastfeeding studies is being able to disentangle the underlying pathways that link breastfeeding to social class. Spanish as well as global previous research shows that mothers with higher socio-economic status or higher level of education breastfeed for longer periods (15–17). Regarding MIS-C, we have included in a clinical case series all children with this diagnosis, admitted to three University hospitals in Catalonia (North–East Spain) during 2020 and 2021. A brief face-to-face questionnaire was used to collect quality data recalled about lactation. We found that only nine out of sixteen children (56%) with MIS-C from our case series had been breastfed at birth (18). The 2017 Spanish Health Survey has reported that eight out of ten Spanish children (80%) are breastfed at hospital discharge (19). According to national standards, Spanish children have about a one-third-better rate of breastfeeding initiation than children with MIS-C in our sample. Hence, the potential protective role of breastfeeding against MIS-C cannot be discarded. But we cannot fail to underline that there are a number of well-known factors that can worsen the clinical picture of COVID-19 (20, 21). In this sense, we must finally admit that the small size of our case series has not allowed us to identify further clinical or demographic characteristics, such as ethnicity, age, overweight, gender, disability, or respiratory pathology, that explain the wide variability of paediatric COVID-19.

Severe COVID-19 is associated with uncontrolled inflammation. Children who were ever breastfed have lower levels of biomarkers that are increased during the cytokine burst (i.e., ferritin, serum monocyte chemoattractant protein-1, or uric acid) than their formula-fed peers (6–9). For this reason, patients who have been breastfed may be in the best position to restore the oxidative balance when they are infected with this virus. In addition, there are likely to be lower rates of prematurity and viral co-infection among children who were breastfed, both risk factors for COVID-19 according to Grazzino et al. In conclusion, there is still a need for a systematic overview of not only recent but also early life characteristics that may influence COVID-19, with emphasis on infant feeding. This would allow researchers and physicians to better understand inequalities in the severity of the disease.
